# Can laparoscopic surgery be applied in gastric gastrointestinal stromal tumors located in unfavorable sites? A study based on the NCCN guidelines

**DOI:** 10.1097/MD.0000000000006535

**Published:** 2017-04-07

**Authors:** Chang-Ming Huang, Qing-Feng Chen, Jian-Xian Lin, Mi Lin, Chao-Hui Zheng, Ping Li, Jian-Wei Xie, Jia-Bin Wang, Jun Lu, Qi-Yue Chen, Long-Long Cao, Ru-Hong Tu

**Affiliations:** Department of Gastric Surgery, Fujian Medical University Union Hospital, Fuzhou, Fujian Province, China.

**Keywords:** gastric gastrointestinal stromal tumor, laparoscopic surgery, open surgery, short-term and long-term outcomes, unfavorable site

## Abstract

This article investigated the feasibility of laparoscopic surgery in unfavorable site gastric gastrointestinal stromal tumors (GISTs).

We identified 214 patients who underwent primary gastric GIST resection at our institution (January 2006–December 2014) from a prospectively collected database. These patients were divided into a Favorable group (140 cases) and an Unfavorable group (74 cases) according to the 2014 version of the National Comprehensive Cancer Network Clinical Guidelines (NCCN guidelines).

The wedge resection rate of the Favorable group was higher than that of the Unfavorable group, and most procedures were performed laparoscopically (*P* < 0.05). In addition, there were no differences in the other clinicopathological features between these groups (*P* > 0.05). The postoperative stay of the Unfavorable group was longer than that of the Favorable group (*P* = 0.02). Laparoscopic surgery in both groups resulted in a shorter operative time, lower blood loss, faster time to first flatus, faster time to first fluid diet, and shorter postoperative stay than open surgery (*P* < 0.05). Although the difference was not significant (*P* = 0.09), the postoperative complication incidence of the Favorable group was less than that of the Unfavorable group (10% vs 17.6%). Furthermore, in the Unfavorable group, the incidence of postoperative complications from laparoscopic surgery was significantly lower than that of open surgery (*P* = 0.001). There were no differences in the 5-year overall survival (OS) and recurrence-free survival (RFS) of these groups (*P* > 0.05). Furthermore, in the Unfavorable group, the 5-year OS and RFS were similar for both laparoscopic and open procedures. Multivariate Cox regression analysis showed that imatinib (IM) treatment was an independent risk factor for poor prognosis.

Laparoscopic operation for gastric GISTs located in unfavorable sites can yield similar long-term outcomes compared with an open operation. However, laparoscopic surgery has the obvious advantage of being minimally invasive, and the incidence of postoperative complications was low. Laparoscopic surgery is thus an option for the treatment of localized gastric GISTs.

## 1 Introduction

The initial treatment for gastrointestinal stromal tumors (GISTs) is surgical resection. Currently, some controversy exists regarding the application of laparoscopic operations for gastric GIST. The 2014 version of the National Comprehensive Cancer Network Clinical Guidelines (NCCN guidelines) ^[1]^ modified the old version regarding the tumor size limitation for laparoscopic surgery and suggests that a laparoscopic approach may be considered for select GISTs in favorable anatomical locations (greater curvature and anterior wall of the stomach) by surgeons with appropriate laparoscopic experience. However, the applicability of laparoscopic surgery in unfavorable sites of gastric GSIT, such as the gastroesophageal junction, the small curvature of the gastric body, and the posterior wall of the stomach, is unclear, and there are few relevant studies. Therefore, this article summarized the clinical data of 214 patients with GISTs in our hospital from January 2006 to December 2014 and analyzed the short- and long-term effects of laparoscopic operations for unfavorable site gastric GISTs.

## 2 Methods

### 2.1 Study population

**Figure 1:**
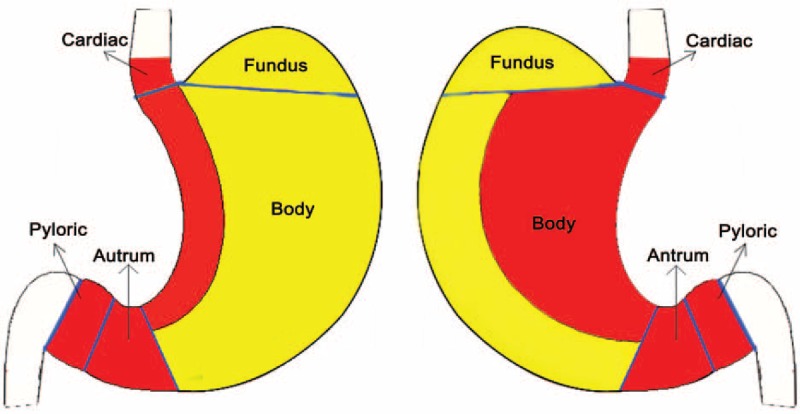
Red: Unfavorable site; yellow: Favorable site.

**Figure 2:**
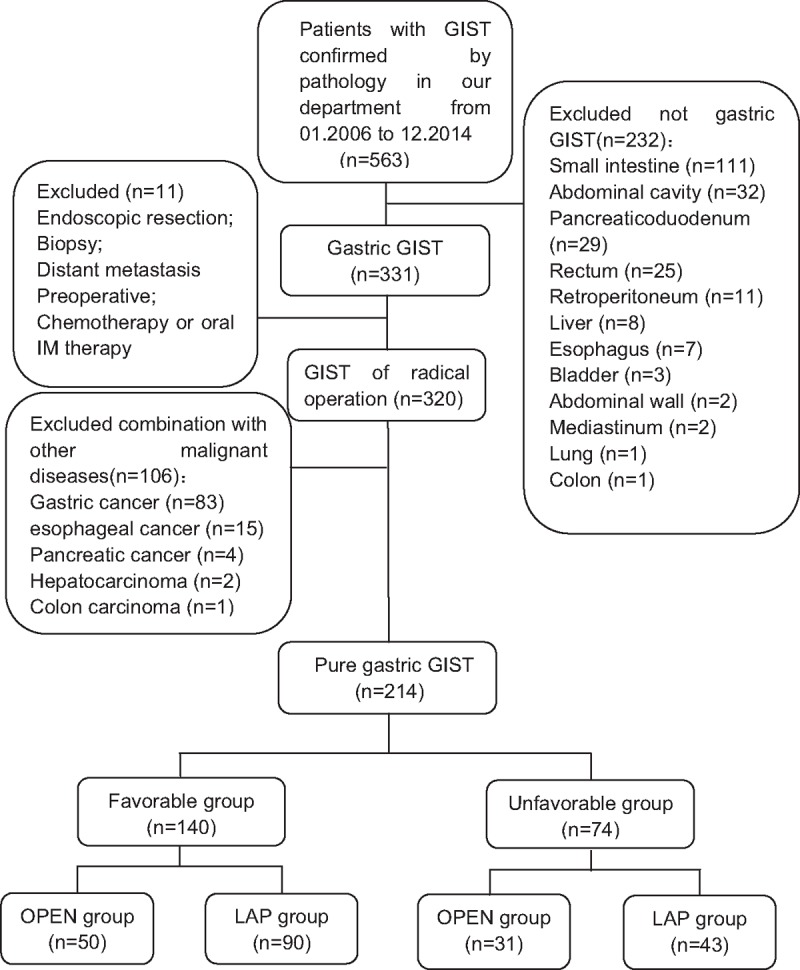
Enrollment of patients in the study.

This study was carried out with the approval of the ethics committee of Fujian Medical University Union Hospital. And the study cohort consisted of 563 GIST (confirmed by pathology) patients at this hospital from January 2006 to December 2014. The primary gastric GIST patients who had not received preoperative chemotherapy or oral imatinib (IM) treatment or previously undergone a radical operation were included in the analysis. We excluded those patients who received endoscopic resection or biopsy or had distant metastasis or the presence of other malignant diseases and those whose tumors were located outside the stomach. Finally, a total of 214 cases were enrolled in the study. This study was conducted in a retrospective way. The choice of surgical methods was based on both the patient's condition and surgeons’ experience. All the 133 cases was conducted laparoscopic surgery successfully, with no one converting to open. These patients were divided into the Favorable group (140 cases) and the Unfavorable group (74 cases) according to the 2014 version of the NCCN guidelines. The tumor sites of the Favorable group were located in the gastric fundus, the anterior wall, and the greater curvature of the gastric body, and this group included 140 cases. The tumor sites in the Unfavorable group were located in the gastroesophageal junction, the lesser curvature of the gastric body, the posterior wall of the stomach, antrum, and pylorus, and this group included 74 cases (Figs. 1 and 2 and Table 1).

**Table 1:**
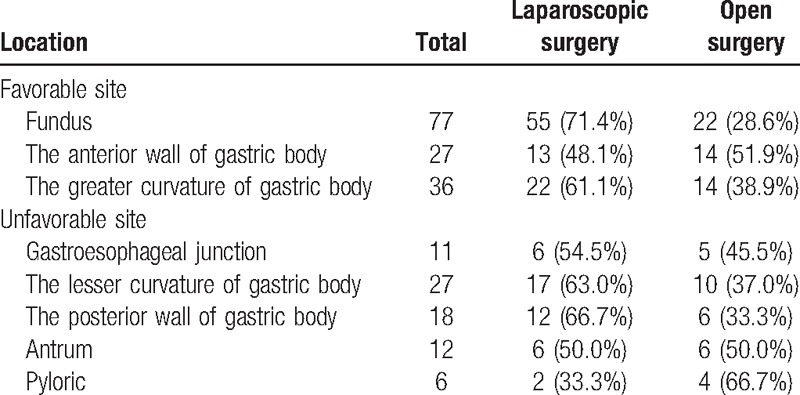
Patient GIST location.

Operation procedure of laparoscopic surgery included the following: The patient was placed in the reverse Trendelenburg position with the head elevated approximately 15° to 20° and tilted left side up at approximately 20° to 30°. The surgeon stood between the patient's legs, with the assistant and camera operator both on the patient's right side. For those whose tumor location or side was unclear before surgery, we would figure out the location or measurement intraoperatively with the help of intraoperative gastroscopy. After the tumor was isolated, we would adopt wedge resection, proximal gastrectomy, and distal gastrectomy or total gastrectomy according to the features of tumor, without touching the tumor directly. Tissue separation, capture, anastomoses were performed in the normal gastric tissue surrounding the tumor. Then, the tumor would be removed from abdominal cavity with a specimen bag.

### 2.2 Variables and definitions

The tumor size was defined as the maximum tumor diameter. The mitotic rates were defined as the number of mitoses per 50 high-power fields. The recurrence risk was graded by the modified National Institutes of Health (NIH) risk-classification scheme.^[2]^ Body mass index (BMI) ≧ 25 was defined as overweight according to the World Health Organization (WHO) criteria. Blood loss was estimated according to the volume of blood absorbed by the gauze and suction pumped after subtracting the volume of fluids used for irrigation. Preoperative comorbidities were classified according to the Charlson comorbidity index (CCI),^[3]^ and postoperative complications were classified according to the Clavien–Dindo classification.^[4]^

### 2.3 Follow-up

Specially trained researchers used outpatient records, visits, letters, and telephone calls to follow-up with the patients postoperatively. The last follow-up period ended in March 2015. The follow-up information included survival status, postoperative review results, tumor recurrence, and/or metastasis and adjuvant therapy. The overall survival (OS) was recorded from the operation time to the end of the follow-up period, the time of death, or the value input in the follow-up database (such as death from other diseases). Recurrence-free survival (RFS) was recorded from surgery to tumor recurrence.

### 2.4 Statistical analyses

All statistical analyses were performed using SPSS version 18.0 (SPSS Inc., Chicago, IL). The measurement data are presented as the means ± standard deviations (SDs). Categorical data were compared with a χ^2^ test or Fisher exact test. The variables with *P* < 0.1 in the univariate analysis were subsequently included in a multivariate binary logistic regression model. The results of the univariate and multivariate analyses were expressed as the odds ratios (ORs) with corresponding 95% confidence intervals (95% CIs). The survival rates were calculated using the Kaplan–Meier method, which used the log-rank test to detect differences in the survival curves of the various subgroups. *P*-values < 0.05 were considered significant.

## 3 Results

### 3.1 Clinicopathological characteristics

The clinical and pathologic variables of the patients are summarized in Table 2. The wedge resection rate of the Favorable group was higher than that of the Unfavorable group, and most of the procedures were performed laparoscopically (*P* < 0.05). There were no differences in the other clinicopathological features between the groups (*P* > 0.05). In the Favorable group, the postoperative IM treatment rate after laparoscopic operation was lower than that after open surgery (37.8% vs 54.0%; *P* = 0.047).

**Table 2:**
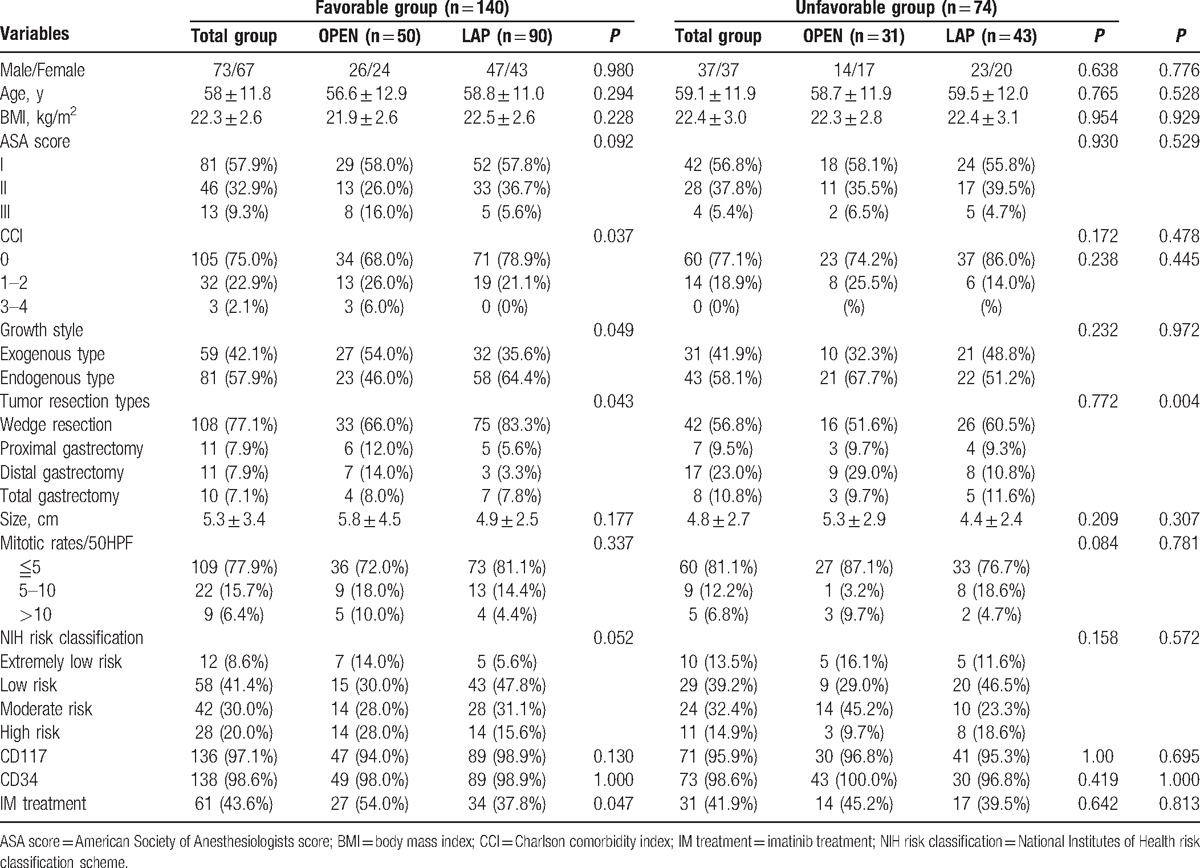
Baseline characteristics of eligible patients.

### 3.2 Perioperative and postoperative results

The mean postoperative stay of the Unfavorable group was significantly longer than that of the Favorable group (9.8 ± 5.4 vs 11.8 ± 7.0 d; *P* = 0.02). In addition, laparoscopic surgery resulted in less blood loss and shorter operation time, time to first flatus, time to first fluid diet, and postoperative stay than open surgery in both patient groups (*P* < 0.05). The perioperative and postoperative results are summarized in Table 3.

**Table 3:**
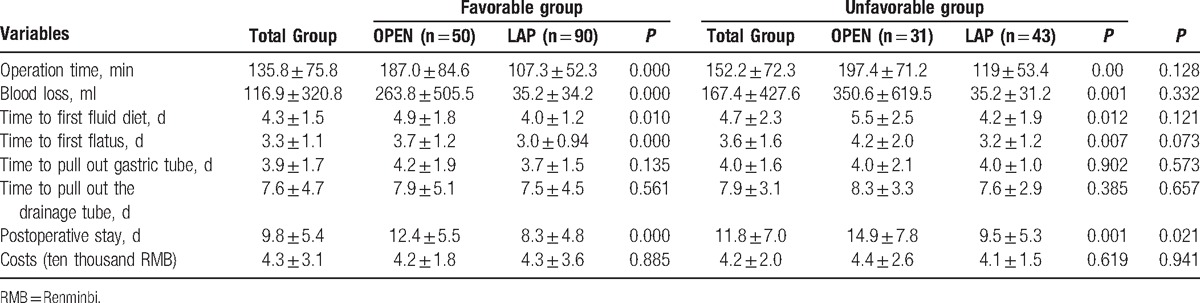
Perioperative and postoperative results.

### 3.3 Postoperative complications

Although this difference was not significant (*P* = 0.09), the postoperative complication incidence of the Favorable group was less than that of the Unfavorable group (10% vs 17.6%). Furthermore, in the Unfavorable group, the incidence of postoperative complications from laparoscopic surgery was significantly less than that of open surgery (*P* = 0.001), especially grade III-IV complications (2.3% vs 29.0%, *P* = 0.001). The postoperative complications are summarized in Table 4.

**Table 4:**
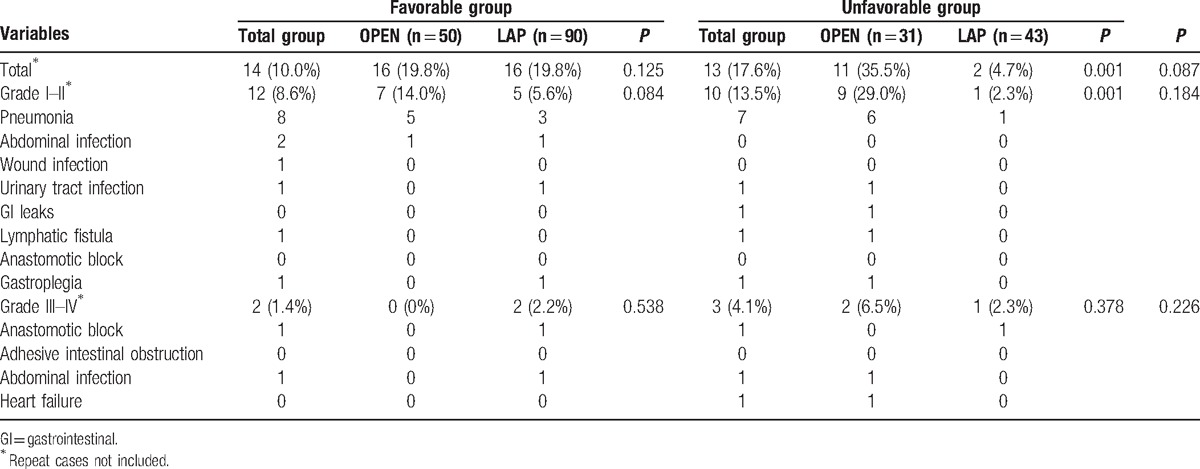
Postoperative complications.

### 3.4 Long-term outcomes

**Figure 3:**
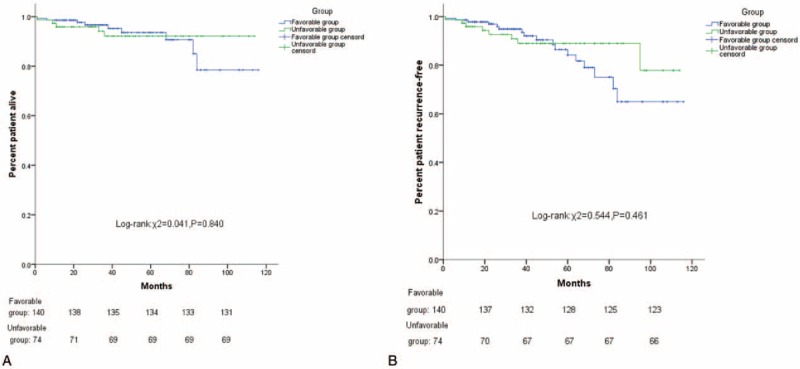
(A) Kaplan–Meier curves of OS stratified by Favorable group versus Unfavorable group (χ^2^ = 0.041, *P* = 0.840). (B) Kaplan–Meier curves of PFS stratified by Favorable group versus Unfavorable group (χ^2^ = 0.544, *P* = 0.461).

**Figure 4:**
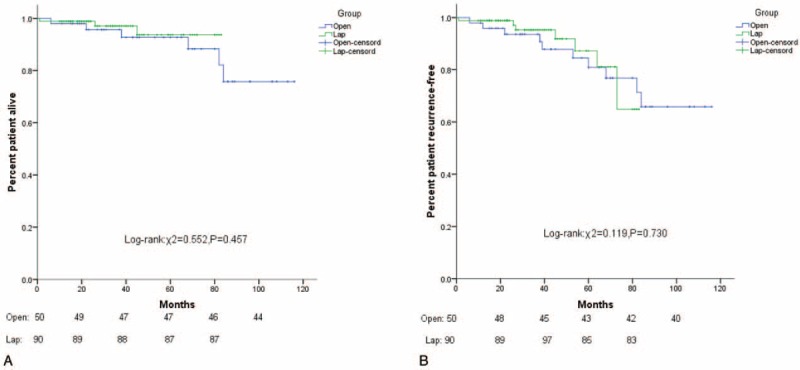
(A) Kaplan–Meier curves of OS stratified by laparoscopic surgery versus open surgery in the Favorable group (χ^2^ = 0.552, *P* = 0.457). (B) Kaplan–Meier curves of PFS stratified by laparoscopic surgery versus open surgery in the Favorable group (χ^2^ = 0.119, *P* = 0.730).

**Figure 5:**
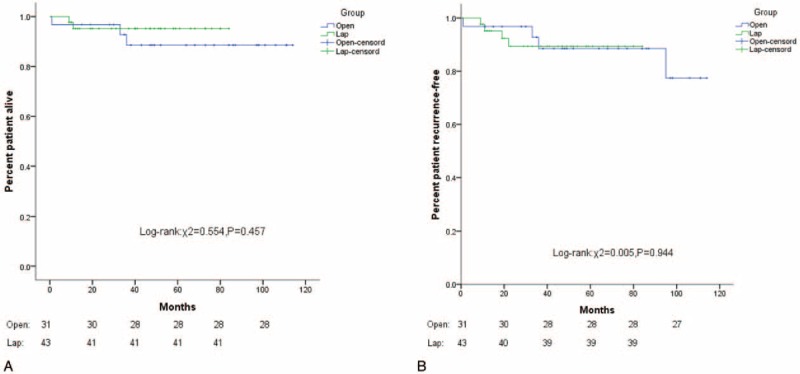
(A) Kaplan–Meier curves of OS stratified by laparoscopic surgery versus open surgery in the Unfavorable group (χ^2^ = 0.554, *P* = 0.457). (B) Kaplan–Meier curves of PFS stratified by laparoscopic surgery versus open surgery in the Unfavorable group (χ^2^ = 0.005, *P* = 0.944).

A total of 201 patients (93.9%) were followed-up. The median follow-up time was 40 months (range: 1–116 months). The 3-year OS values in the Favorable group and the Unfavorable group were 96.6% and 92.1%, respectively. The 5-year OS values were 90.7% and 92.1%, respectively (*P* = 0.84). The 3-year RFS values of the Favorable group and Unfavorable group were 95.8% and 90.5%, respectively. The 5-year RFS values were 84.2% and 89.0%, respectively (*P* = 0.46) (Fig. 3A, B). Furthermore, in both groups, the 5-year OS and 5-year RFS of the laparoscopic surgery and open surgery were similar (*P* > 0.05) (Figs. 4A, B and 5A, B).

### 3.5 Univariate and multivariate analyses of OS

A univariate Cox regression analysis showed that preoperative albumin (ALB), American Society of Anesthesiologists (ASA) score, CCI, tumor size, mitotic rates, tumor resection methods, and IM treatment were pretty related to the 5-year OS. Multivariate analysis showed that IM treatment was an independent factor for patients’ prognosis [hazard ratio (HR) = 0.328, *P* = 0.007]. Furthermore, in high-risk groups, patients who took IM treatment after surgery had a higher rate of 5-year RFS than those who did not (74.8% vs 68.3%, *P* = 0.034) (Table 5).

**Table 5:**
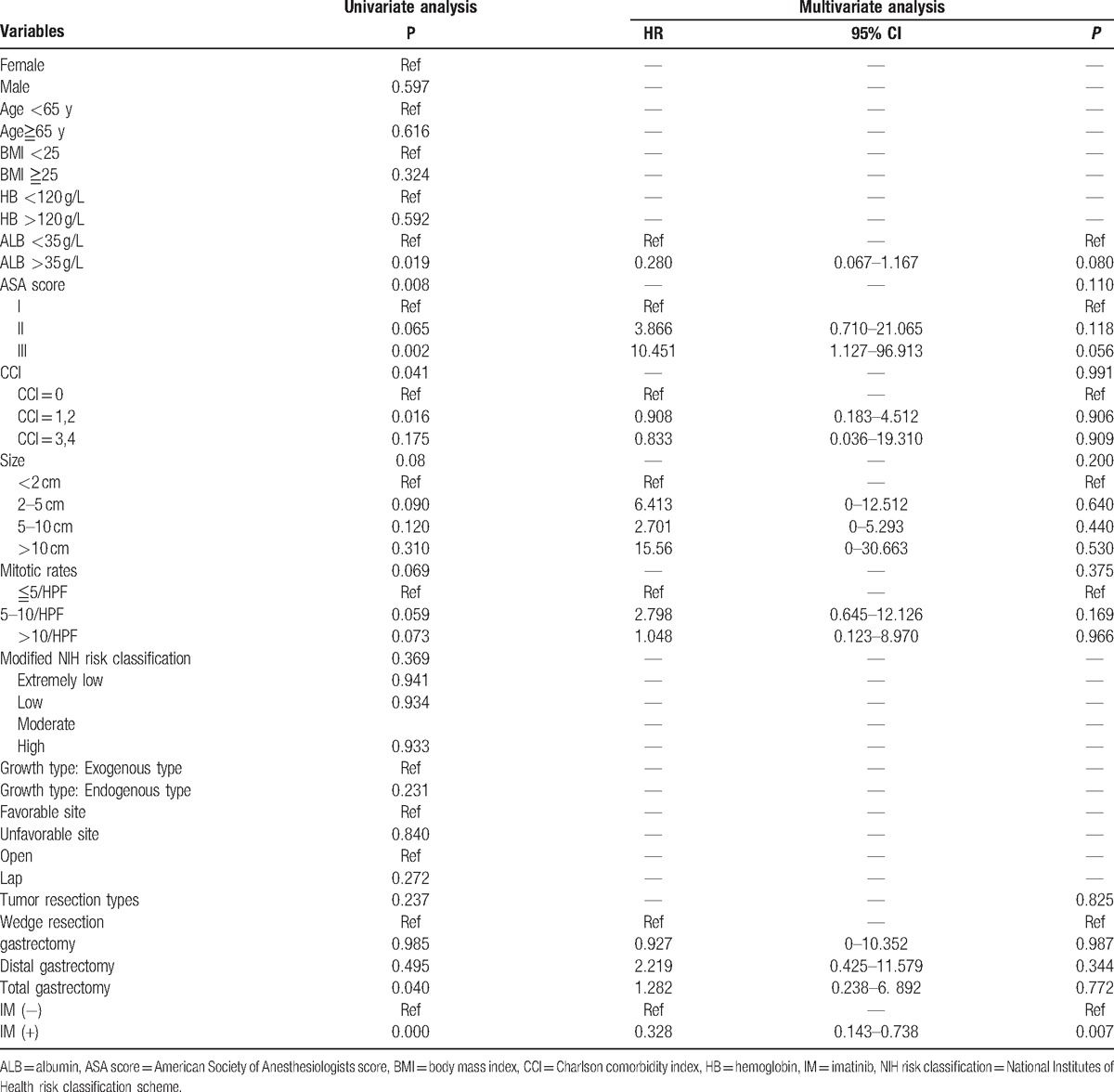
Cox analysis of 5-year OS.

## 4 Discussion

The stomach is the most common site for GISTs and accounts for 50% to 70% of cases.^[5–7]^ Surgical operation is the primary treatment for gastric GISTs. The application of laparoscopic surgery for gastric GISTs is rapidly increasing.^[8–20]^ Masoni et al^[16]^ excluded patients whose tumors were located at the gastroesophageal junction and pylorus and analyzed 24 patients who underwent laparoscopic operation. The results showed that no patients had major complications and that no recurrence occurred after 75 months of follow-up. Karakousis et al^[8]^ enrolled a total of 80 patients after excluding 14 patients whose tumors were located in the gastroesophageal junction. The results showed that laparoscopic surgery reduced the intraoperative bleeding and postoperative hospital stay. In addition, in patients whose tumor was located in a favorable site in our study, laparoscopic surgery shortened the operation time, reduced intraoperative bleeding, and accelerated the recovery of gastrointestinal function after surgery compared with open surgery. Laparoscopic surgery for gastric GISTs located in the favorable site has the clear advantage of being minimally invasive. Therefore, the 2014 version of the NCCN guideline suggested that the laparoscopic approach be considered for select GISTs in favorable anatomical locations (e.g., the greater curvature of the stomach or anterior wall of the stomach).

However, research on laparoscopic operations in unfavorable site gastric GISTs is lacking. Some scholars believe that gastric GIST locations are too deep to expose when the tumor is located in the gastroesophageal junction, the lesser curvature, and the posterior wall of the gastric body, which increases the surgical difficulty.^[13,15,21,22]^ Nguyen et al^[21]^ analyzed 28 cases of gastric GIST patients with attempted laparoscopic surgery. In that study, 3 cases were converted to open surgery, and 2 were attributed to a tumor near the gastroesophageal junction. In the study by Poškus et al,^[22]^ the conversion rate of gastric GISTs located at the gastroesophageal junction and cardia was 100% (4 cases were converted to open surgery in total). Therefore, laparoscopic surgery is not recommended for gastric GISTs in those anatomical locations in the 2014 version of the NCCN guidelines. This article analyzed 74 cases with tumors located in unfavorable sites and represents the largest number of cases studied with regard to the short-term and long-term outcomes of laparoscopic surgery for unfavorable site gastric GISTs to date. We found that in the Unfavorable group, laparoscopic surgery significantly shortened the operation time, reduced intraoperative bleeding, shortened the time to first flatus and the time to first fluid diet, and reduced the incidence of postoperative complications, suggesting that laparoscopic operation can provide the patients with better short-term outcomes than open surgery. We believe that when the tumor is located in an unfavorable site, the field of view and the visualization of anatomical structures are improved under laparoscopic operation, thereby reducing unnecessary tissue or vascular injury. In addition to the small incision, laparoscopic surgery can also shorten the operation time and reduce surgical trauma and the incidence of postoperative complications.

The longest follow-up period in our research was 116 months. In both the Favorable and Unfavorable groups, laparoscopic surgery and open surgery had similar prognoses. Furthermore, the 5-year OS determined by Cox regression analysis revealed that only postoperative IM treatment is an independent risk factor for poor prognosis. In contrast, the GIST anatomical site and surgical method did not affect long-term outcomes of gastric GIST patients. However, in the Favorable group, the postoperative IM treatment rate after laparoscopic operation was lower than that after open surgery. In our opinion, the number of patients in extremely low risk and low risk in the laparoscopic group were significantly less than that in the open group (47.8% vs 30%, *P* = 0.052) owing to the certain selection biases, which reduced the treating rate of IM after laparoscopic operation. In conclusion, compared with open operation, laparoscopic operation for gastric GISTs located in unfavorable sites can yield similar long-term outcomes. However, laparoscopic operations have the obvious advantage of being minimally invasive with a low incidence of postoperative complications. Therefore, laparoscopic surgery is an option for the treatment of localized gastric GISTs. The analysis of this study was based on the retrospective data of a single center. And, the choice of surgical methods was based on both the patient's condition and surgeons’ experience in this retrospective study, which inevitably led to some certain selection bias. Also, it is a main reason for that no any conversion to open surgical procedure occurred in this research. Therefore, to improve the accuracy and reliability of the study, it needs a larger scale, prospective, multicenter randomized controlled study.

## References

[1] von Mehren M Randall RL Benjamin RS Gastrointestinal stromal tumors, version 2.2014. J Natl Compr Canc Netw 2014;12:853–62. 24925196 10.6004/jnccn.2014.0080

[2] Joensuu H Risk stratification of patients diagnosed with gastrointestinal stromal tumor. Hum Pathol 2008;39:1411–9. 18774375 10.1016/j.humpath.2008.06.025

[3] Charlson ME Pompei P Ales KL A new method of classifying prognostic comorbidity in longitudinal studies: development and validation. J Chronic Dis 1987;40:373–83. 3558716 10.1016/0021-9681(87)90171-8

[4] Dindo D Demartines N Clavien P Classification of surgical complications: a new proposal with evaluation in a cohort of 6336 patients and results of a survey. Ann Surg 2004;240:205–13. 15273542 10.1097/01.sla.0000133083.54934.ae PMC1360123

[5] Pidhorecky I Cheney RT Kraybill WG Gastrointestinal stromal tumors: current diagnosis, biologic behavior, and management. Ann Surg Oncol 2000;7:705–12. 11034250 10.1007/s10434-000-0705-6

[6] Demetri GD von Mehren M Antonescu CR NCCN Task Force report: update on the management of patients with gastrointestinal stromal tumors. J Natl Compr Canc Netw 2010;8 (suppl 2):S1–41. 10.6004/jnccn.2010.0116 PMC4103754 20457867

[7] Miettinen M Majidi M Lasota J Pathology and diagnostic criteria of gastrointestinal stromal tumors (GISTs): a review. Eur J Cancer 2002;38 (suppl 5):S39–51. 10.1016/s0959-8049(02)80602-5 12528772

[8] Karakousis GC Singer S Zheng J Laparoscopic versus open gastric resections for primary gastrointestinal stromal tumors (GISTs): a size-matched comparison. Ann Surg Oncol 2011;18:1599–605. 21207158 10.1245/s10434-010-1517-y PMC4986692

[9] Melstrom LG Phillips JD Bentrem DJ Laparoscopic versus open resection of gastric gastrointestinal stromal tumors. Am J Clin Oncol 2012;35:451–4. 21552096 10.1097/COC.0b013e31821954a7

[10] Koh Y Chok A Zheng H A systematic review and meta-analysis comparing laparoscopic versus open gastric resections for gastrointestinal stromal tumors of the stomach. Ann Surg Oncol 2013;20:3549–60. 23793362 10.1245/s10434-013-3051-1

[11] Ganai S Prachand VN Posner MC Predictors of unsuccessful laparoscopic resection of gastric submucosal neoplasms. J Gastrointest Surg 2013;17:244–55. 23225195 10.1007/s11605-012-2095-z

[12] De Vogelaere K Hoorens A Haentjens P Laparoscopic versus open resection of gastrointestinal stromal tumors of the stomach. Surg Endosc 2013;27:1546–54. 23233005 10.1007/s00464-012-2622-8

[13] Bischof DA Kim Y Dodson R Open versus minimally invasive resection of gastric GIST: a multi-institutional analysis of short- and long-term outcomes. Ann Surg Oncol 2014;21:2941–8. 24763984 10.1245/s10434-014-3733-3

[14] Lin J Huang C Zheng C Laparoscopic versus open gastric resection for larger than 5 cm primary gastric gastrointestinal stromal tumors (GIST): a size-matched comparison. Surg Endosc 2014;28:2577–83. 24853837 10.1007/s00464-014-3506-x

[15] Honda M Hiki N Nunobe S Long-term and surgical outcomes of laparoscopic surgery for gastric gastrointestinal stromal tumors. Surg Endosc 2014;28:2317–22. 24566748 10.1007/s00464-014-3459-0

[16] Masoni L Gentili I Maglio R Laparoscopic resection of large gastric GISTs: feasibility and long-term results. Surg Endosc 2014;28:2905–10. 24879133 10.1007/s00464-014-3552-4

[17] Hsiao C Yang C Lai I Laparoscopic resection for large gastric gastrointestinal stromal tumor (GIST): intermediate follow-up results. Surg Endosc 2015;29:868–73. 25052129 10.1007/s00464-014-3742-0

[18] Chen K Zhou Y Mou Y Systematic review and meta-analysis of safety and efficacy of laparoscopic resection for gastrointestinal stromal tumors of the stomach. Surg Endosc 2015;29:355–67. 10.1007/s00464-014-3676-6 25005014

[19] Chen K Zhou YC Mou YP Systematic review and meta-analysis of safety and efficacy of laparoscopic resection for gastrointestinal stromal tumors of the stomach. Surg Endosc 2015;29:355–67. 10.1007/s00464-014-3676-6 25005014

[20] Dressler JA Palazzo F Berger AC Long-term functional outcomes of laparoscopic resection for gastric gastrointestinal stromal tumors. Surg Endosc 2016;30:1592–8. 26169640 10.1007/s00464-015-4384-6

[21] Nguyen SQ Divino CM Wang J Laparoscopic management of gastrointestinal stromal tumors. Surg Endosc 2006;20:713–6. 16502196 10.1007/s00464-005-0435-8

[22] Poškus E Petrik P Petrik E Surgical management of gastrointestinal stromal tumors: a single center experience. Wideochir Inne Tech Maloinwazyjne 2014;9:71–82. 24729813 10.5114/wiitm.2014.40987 PMC3983553

